# Leucine Regulates Zoosporic Germination and Infection by *Phytophthora erythroseptica*

**DOI:** 10.3389/fmicb.2019.00131

**Published:** 2019-02-05

**Authors:** He Jiang, Hye Weon Hwang, Tongling Ge, Barbara Cole, Brian Perkins, Jianjun Hao

**Affiliations:** ^1^School of Food and Agriculture, The University of Maine, Orono, ME, United States; ^2^Department of Chemistry, The University of Maine, Orono, ME, United States

**Keywords:** oomycetes, quorum sensing, amino acid, plant pathogen, zoospore

## Abstract

Pink rot (*Phytophthora erythroseptica*) of potato is a major concern in many potato production regions. The pathogen produces zoospores that serve as a primary inoculum for infection. To understand how the pink rot incidence is related to pathogen population, qualitative, and quantitative chemical analyses were conducted. It was demonstrated that *P. erythroseptica* zoospores required a minimal population of 10^3^ zoospores/ml (threshold) for initiating germination and the subsequent infection; the percentage of zoosporic germination was positively correlated with the density of zoospores above the threshold. To elucidate the density-dependent behavior, zoospore exudate (ZE) was extracted from high-density (10^5^/ml) zoospore suspension. Zoosporic inocula of *P. erythroseptica* at different concentrations were inoculated on potato tubers. Necrotic lesions were caused by inoculum with 100 zoospores per inoculation site; 5 zoospores per site did not cause lesions on the tuber. However, five zoospores did cause lesions when they were placed in ZE, suggesting ZE contained chemical compounds that regulate germination of zoospores. ZE was collected and analyzed using liquid chromatography mass spectroscopy (LC-MS). Results showed that the amino acid leucine was associated with zoosporic germination. Therefore, zoosporic germination and infection of *P. erythroseptica* were mediated by signaling molecules secreted from zoospores.

## Introduction

*Phytophthora erythroseptica* Pethyb., the causal agent of pink rot of potato (*Solanum tuberosum*), is a major threat to the potato industry throughout North America, including the state of Maine (Lambert and Salas, 1994; Taylor et al., 2002; Fitzpatrick-Peabody, 2011). It can cause significant yield loss of potato at both field and storage stages (Vargas and Nielsen, 1972; Peters et al., 2001; Taylor et al., 2011). In controlling pink rot, the most effective method is the use of chemicals, such as mefenoxam and phosphorous acid (Chapara et al., 2007; Taylor et al., 2011). However, extensive use of chemicals can result in loss of efficacy due to the fast development of resistant populations of *P. erythroseptica* in the field (Chapara et al., 2011; Fitzpatrick-Peabody, 2011).

*Phytophthora erythroseptica* produces oospores and sporangia for reproduction (Pratt, 1981). Sporangia produce and release multiple zoospores under optimal conditions to initiate infection on the host plant (Erwin and Ribeiro, 1996). The uninucleate zoospore has two anisokont flagella, which facilitate swimming. Zoospores encyst to start infection once they arrive at the host surface (Erwin and Ribeiro, 1996). As a primary inoculum, zoospores are more effective at initiating disease than sporangia or mycelia (Lonsdale et al., 1980).

Zoospores of *P. erythroseptica* have multiple pre-infection stages. Motile zoospores migrate toward and accumulate at the host area. The encysted zoospores adhere on the host surface and germinate for the penetration into host cell (Deacon and Donaldson, 1993). Zoospores are stimulated by several signaling compounds from the host, environmental microorganisms, or themselves for site selection, aggregation, encystment, germination, and penetration through chemotaxis or electrotaxis (Savory et al., 2014; Suo et al., 2016). The signaling molecules may be amino acids, sugars, aldehydes, isoflavones, and alcohols (Deacon and Donaldson, 1993; Morris et al., 1995; Suo et al., 2016). More importantly, the responses of *Phytophthora* zoosporic behaviors to the signaling molecules depend on zoospore density (Kong and Hong, 2010; Jiang and Hao, 2015). For example, a single spore can rarely germinate even under favorable condition and therefore cannot initiate disease (Clarke, 1966). Zoospores of *P. capsici, P. infestans, P. nicotianae, P. sojae*, and *P. palmivora* only aggregate when the spore concentration is higher than 10^5^–10^6^/ml (Ko and Chan, 1974; Kong and Hong, 2010; Kong et al., 2010b). Kong et al. (2010b) illustrated that some extracellular compounds produced by zoospores are required and they enhanced both host targeting and infection. In addition, the signal compounds may be shared among related species of oomycetes and other soil bacteria, but the molecules are yet to be identified (Kong and Hong, 2010, 2016; Kong et al., 2010b).

Although the chemical compounds serving as signaling molecules are not fully understood or identified in limited studies in oomycetes, similar phenomena have been widely reported in bacteria and some true fungi. In these systems, chemical compounds that have been identified as signal molecules include oligopeptides, autoinducer-2, and γ-butyrolactone [N-Acyl homoserine lactones (AHLs) and A-factor] in bacteria (Fuqua et al., 2001; Kong et al., 2010a); and γ-butyrolactone, multicolic acid in filamentous fungi (Raina et al., 2010, 2012). Those signal compounds are involved in important processes of both bacteria and fungi (Fuqua et al., 2001; Hornby et al., 2001; Kong et al., 2010b; Raina et al., 2012). Accumulated signals can activate specific receptors in bacteria, which will regulate transcription of many important genes that are related to biofilm formation, colonization, and host infection (Hirsch et al., 2003; Li and Nair, 2012). Hornby et al. (2001) first observed signal-regulated behaviors in dimorphic fungus *Candida albicans* as the main factor that regulated germ tube formation. After that, more signal molecules were identified in *Penicillium sclerotiorum* and *Aspergillus terreus* (Raina et al., 2010, 2012). Based on current data, oomycetes may not use the same chemicals in quorum sensing activities (Kong et al., 2010a), and therefore further studies are required.

In *P. erythroseptica*, there is no documentation of the chemical regulation of zoospores. Our preliminary results demonstrated that zoosporic germination was dependent on zoospore concentration (Jiang and Hao, 2015). We hypothesized that zoosporic behaviors of *P. erythroseptica*, in response to extracellular signaling compounds, are regulated through a density-dependent way. The goal of this study was to investigate zoosporic behaviors of *P. erythroseptica* mediated by density-related signaling molecules and understand the correlation between disease initiation and pathogen density. In a long-term consideration, this finding will help to improve disease management.

## Materials and Methods

### Zoospore Production by *P. erythroseptica*

*Phytophthora erythroseptica* was isolated from infected potato tubers in Maine in 2013. The isolates were confirmed by sequence analysis using internal transcribed spacer 1 region of rDNA. The culture was maintained on V8 agar Erwin and Ribeiro, 1996) throughout the study. Preparation of zoospore suspension was conducted based on the methods described by others (Fitzpatrick-Peabody, 2011) with slight modification. Briefly, mycelial plugs on 2% V8 agar medium were placed in 3% lima bean broth and incubated 3 days at 22°C in the dark for vegetative growth. For sporangial production, the mycelial mats were transferred to 10% soil extract solution after rinsing with sterile distilled water. To maintain a low nutrient condition, the seedling plugs were removed before being incubated at 18°C for 4 days under fluorescent light. Zoospores could be harvested after the mycelial mats were flooded with chilled sterile distilled water at 4°C for 1 h. Zoospore suspension was achieved after filtration through one layer of Miracloth to remove mycelium, sporangia, and other structures. The concentration was determined using a hemocytometer.

### Preparation of Zoospore Exudates

Zoospore exudate (ZE) was defined as the exudate from zoospore suspension at a concentration of >5 × 10^5^ zoospores/ml. To test the effects of ZE on zoosporic behaviors, ZE must be separated from zoospores. The zoospore suspension obtained as described above was filtered through a 0.2-μm filter membrane to remove zoospores and cysts. The filtrate was used to investigate its effects on zoosporic germination and infection. To confirm that the exudates come from zoospores instead of other *P. erythroseptica* structures, liquid culture exudates at different stages, including exudates before chilling and zoospore release, were collected for use in the experiments described below.

### Assay of Zoosporic Germination and Aggregation

The method of Kong and Hong (2010) was used for the zoosporic germination assay with slight modification. Zoospore suspension of *P. erythroseptica* at a concentration of 5 × 10^5^ zoospores/ml was diluted to 5 × 10^2^ spores/ml using either tested solution or sterile distilled water and incubated at 22°C on depression well slides. The tested solutions include ZE from 5 × 10^5^ spores/ml suspension and its dilution series, liquid exudates of *P. erythroseptica* at different stages: 0 and 1 h after chilling, before and after zoospore release; dilution series of V8 broth, lima bean broth, and CaCl_2_ solution (concentration at 0.01, 0.1, 1, 10 mM). Sterile distilled water was used as control. The original zoospore suspension without dilution was used as positive control. The zoospore suspension on depression wells were incubated at 22°C in 10 cm Petri plates over a water-soaked filter paper to maintain the moisture. After 4 h of incubation, zoospore germination was examined for 50 spores from each well. Zoosporic aggregation was recorded not only at different spore concentration but also at multiple temperatures, including 4, 15, 18, and 22°C. The experiment was conducted twice.

### Assay of Zoosporic Infection on Potato Tuber

To determine the correlation between zoospore density and host infection, two trials were conducted. In the first trial, healthy “Russet Norkotah” (pink rot susceptible variety) tubers were surface disinfested with 0.6% sodium hypochlorite. Three eyes on each tuber were inoculated with 10 μl of zoospore suspension at different densities: 0, 1, 5, 10, 20, 50, 100, or 600 zoospores per site. Three tubers were tested for each treatment. The tubers treated with sterile distilled water were used as control. The treated tubers were incubated at 22°C on top of a plastic grid positioned over a water-soaked paper towel to maintain the moisture. After 8 days of incubation, the treated tubers were cut and exposed to the air for 15–20 min, resulting in the infected tuber tissues turn into pink color, which is a typical symptom caused by *P. erythroseptica*. In the second trial, surface disinfested tubers were vertically cut into 5 mm thick slices. Zoospore suspension was diluted with either ZE or sterile distilled water to different concentrations. Ten microliters of diluted zoospore suspension were inoculated onto the potato tuber slice at various densities as above. Five tuber slices were tested for each treatment. The tubers treated with sterile distilled water were used as control. The treated tubers were incubated at 22°C on top of a plastic grid positioned over a water-soaked paper towel to maintain the moisture. After 6 days of incubation, the treated tuber slices were examined for necrotic lesions. The experiments were conducted twice.

### Comparison of ZE and Other Chemicals on Zoosporic Germination

To confirm whether *P. erythroseptica* zoosporic germination was induced by Ca^2+^ in ZE, CaCl_2_ solution at different concentrations was used to treat zoospore suspension at 1 × 10^3^ spores/ml. After 4-h incubation, the zoosporic germination ratio was evaluated. Sterile water was used as negative control, and ZE from zoospore suspension at 1 × 10^5^ spores/ml was used as positive control. To confirm whether the nutrient residue in ZE may contribute to zoosporic germination, diluted V8 and lima bean broth were also used to treat zoospores. The germination results were compared with that from ZE treatment.

### Characterization of ZE

Zoospore exudate was examined for its activity to various factors, including multiple temperature and enzymes. To test the effect of temperature, 10 ml ZE was incubated in a 15-ml tube, which was placed in water bath (VWR Scientific Inc., Radnor, PA, United States) at temperatures of 40, 60, 80, and 100°C for 20 min, or in an autoclave (Amsco Lab 250 sterilizer, Steris Inc., Mentor, OH, United States) at 121°C for 15 min. Proteinase K (Sigma-Aldrich, Inc., St. Louis, MO, United States, 37°C, pH 7.5), and catalase (MP Biomedicals, 25°C, pH 7.0) were tested for their effect on activity by incubating ZE with 10 mg/ml of each enzyme for 2 h at the optimal temperature according to the manufacturer’s instruction. Zoospore germination and tuber infection were tested using treated ZE as described above. Enzymes and solvents only were tested at the same time as negative controls. Four replicates were set for each treatment, and the experiments were conducted twice.

### Identification of Signal Compounds in ZE

To prepare the samples for high performance liquid chromatography (HPLC) analysis, 200 ml ZE harvested from a high-density zoospore suspension was concentrated using a lyophilizer (Labconco, Kansas City, MO, United States). The residual powder was dissolved in 500 μl HPLC grade water. Prior to HPLC analysis, the samples were centrifuged at 12,000 *g* for 10 min and the supernatant was filtered through a 0.45 μm syringe filter (PTFE; 13 mm; 0.45 μm pore size; Fisher Scientific, Pittsburgh, PA, United States). Samples were analyzed using an Agilent 1100 series HPLC (Agilent Technologies, Santa Clara, CA, United States) coupled with an auto-sampler, a column thermostat, and a diode-array detector (DAD). Chromatographic separations were performed on a NH_2_ column with a particle size of 5 μm Hypersil (4.6 mm × 250 mm, Phenomenex, Torrance, CA, United States). For gradient elution, mobile phase A (water) and B (acetonitrile) were employed. The following gradient was used: 0–5 min, 90% A, 10% B; 5–15 min, from 90% A, 10% B to 50% A, 50% B, linear gradient; 15–25 min, 50% A, 50% B; 25–30 min, from 50% A, 50% B to 90% A, 10% B, linear gradient; 30–40 min, 90% A, 10% B. The sample injection volume was 40 μl. The flow rate was 1 ml/min, and the column temperature was 25°C. Compounds were separated and detected by retention times and UV spectra (230 nm wave). The components of ZE were collected separately at different retention times from the HPLC. Water only was injected as control. The eluate fractions were collected at the same retention time point. The collected liquid was concentrated and dissolved in HPLC grade water and their effect on zoosporic germination was tested as described above. The HPLC fractions that had signal activity were further identified using liquid chromatography-mass spectrometry (LC-MS). Chromatographic separations were performed on a Kintex C18 column (4.6 mm × 150 mm) with a particle size 5 μm, equipped with a 4.6 mm × 12 mm guard column (Phenomenex, Torrance, CA, United States). The column compartment was maintained at 25°C. The flow rate was 0.4 mL/min, and the injection volume was 40 μL. For gradient elution, mobile phase A (0.1% formic acid) and B (acetonitrile with 0.1% formic acid) were employed. The following gradient was used: 0–5 min, 90% A, 10% B; 5–30 min, from 90% A, 10% B to 50% A, 50% B, linear gradient; 30–35 min, from 50% A, 50% B to 90% A, 10% B, linear gradient. Instrument operation and data analysis were performed using the Agilent LC-MSD Chemstation software.

Further identification was conducted by LC-MSD-ES ion trap mass spectrometry. The ion trap was operated using the ESI source in positive mode to focus on compounds producing a pseudo-molecular ion at *m/z* 50–500. The ion charge control target was set to 10,000 with a maximum accumulation time of 200 min. The MS conditions had been optimized with a capillary temperature of 350°C, a dry gas (nitrogen) flow rate of 9 L/min, and a nebulizer pressure with helium gas of 50 psi. The capillary voltage and trap driver voltage were maintained at 84 and 36.5 V, respectively.

### Statistical Analysis

Data were analyzed using JMP statistical software (version 9.0, SAS Institute Inc., Cary, NC, United States). Analysis of variance (ANOVA) was conducted to analyze treatment effects. Mean separation was performed using Fisher’s protected least significant difference. An alpha level of 0.05 was used for all analysis. In analyzing the relationship between zoosporic germination and the concentration of ZE dilution factors, regression was performed by combining the data of two independent trials, with each having three replicates. All the experiments were conducted twice. If there was no interaction between repeated trials (*P* > 0.05), data were combined from all trials.

## Results

### Correlation Between Density and Behavior of Zoospores

**FIGURE 1:**
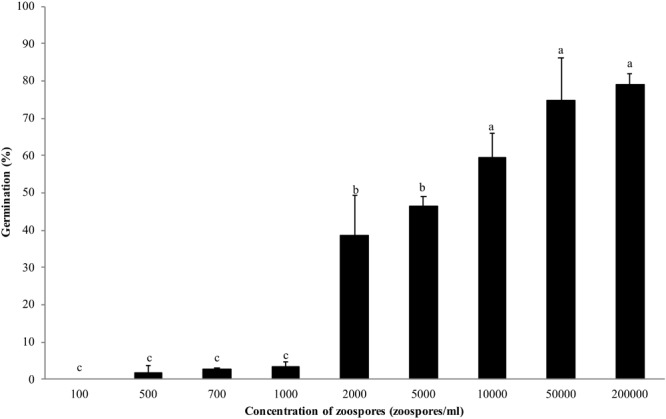
Germination of *Phytophthora erythroseptica* zoospores at various concentrations. Each bar represents the mean ± standard deviation. Means followed by different letters are significantly different (*P* < 0.05) according to Fisher’s protected least significant difference.

**FIGURE 2:**
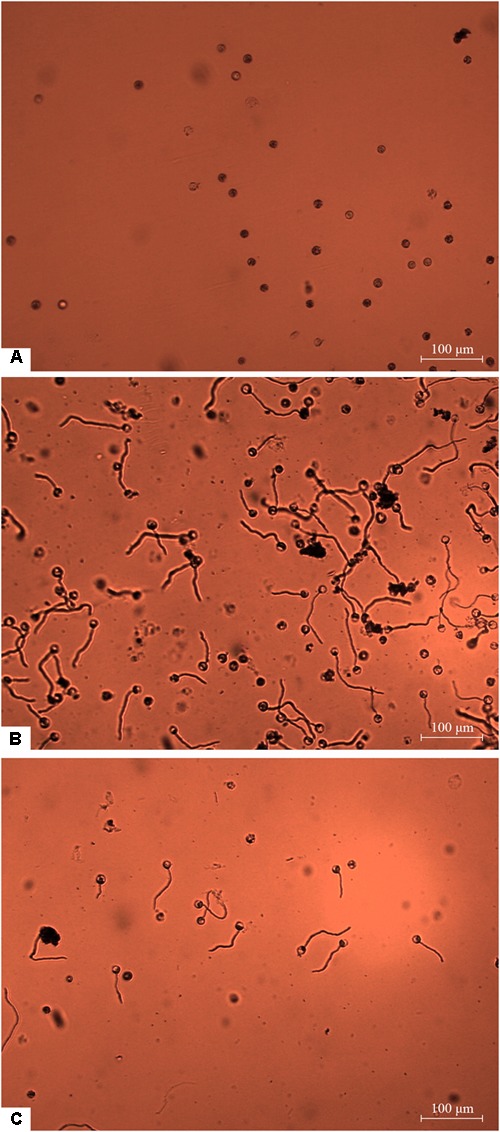
Germination of *Phytophthora erythroseptica* zoospores. The germination was examined under microscope at 100× magnification at 4 h after treatment. The germ tube longer than half of the spore diameter was considered as germinated. No zoospores germinated at concentration of 1 × 10^3^ spores/ml **(A)**. Zoospores germinated at 1 × 10^4^ spores/ml **(B)**. Zoospores germinated at 1 × 10^3^ spores/ml when suspended in zoospore exudate (ZE) derived from zoospore suspension (1 × 10^5^ spores/ml) **(C)**.

**FIGURE 3:**
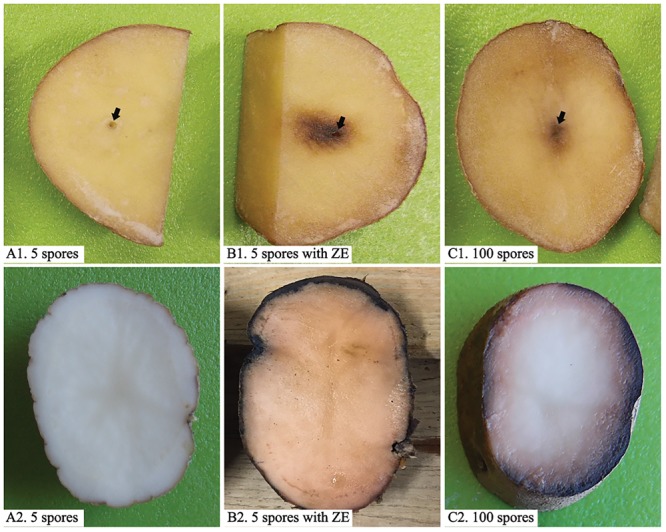
Inoculation of potato tubers (cv. “Russet Norkotah”) with *Phytophthora erythroseptica* zoospores. Either tuber slices (upper panels) or whole tubers (lower panels) of potato were inoculated with 5 zoospores suspended in 10 μl sterile distilled water **(A1,A2)**, 5 zoospores suspended in 10 μl exudate derived from high-density zoospore suspension (1 × 10^4^ spores/ml) **(B1,B2)**, and 100 zoospores suspended in 10 μl sterile distilled water **(C1,C2)**. Arrows indicate inoculation position.

Zoospores did not germinate at concentrations of ≤1 × 10^3^ zoospores/ml, but germination was observed when the concentration was equal to or higher than 2 × 10^3^ spores/ml (Figure 1, 2A,B), where the frequency of germination increased as the concentration of zoospores increased (Figure 1). No specific aggregation was observed at any concentrations or temperatures. For the tuber infection assay, necrotic lesions were obtained only on the tuber slices treated with 100 or more zoospores (Figure 3C1). Tuber slices treated with 100 zoospores had disease incidence as high as 100% (*P* < 0.05). The same results were observed in the whole tuber infection test (Figure 3C2). Zoosporic infection results were correlated with germination, which illustrated that germination is more critical during zoosporic host infection compared with auto-aggregation for *P. erythroseptica*.

### Effect of ZE and Other Chemicals on Zoosporic Germination

**FIGURE 4:**
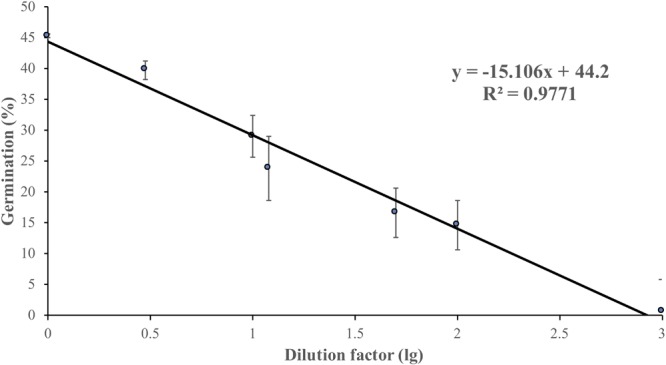
Zoosporic germination of *Phytophthora erythroseptica* versus zoospore exudate (ZE) dilution factors. ZE extracted from 5 × 10^5^ spores/ml was serially diluted by the dilution factors (*X*-axis). A linear regression between dilution factor (*X*) and germination (*Y*) was established as follows: Y = -6.61X + 44.11, R^2^ = 0.625. Each data point was the average of two independent trials with three replicates in each trial. The standard error of the coefficient of *X* is 0.72, and the coefficient is significant at the 1% level. The standard error of the coefficient of the constant term is 2.51, and the coefficient is also significant at the 1% level.

Non-treated zoospores of *P. erythroseptica* germinated at 2 × 10^3^ spores/ml or higher densities (Figure 1). However, when treated with ZE, zoospores at lower concentrations (<5 × 10^2^ spores/ml) germinated (Figure 2C). The germination rate was as high as that of zoospores at 2 × 10^3^ spores/ml or higher concentration (Figure 4). When ZE was serially diluted, the induced effects declined as the dilution factor increased (Figure 4). ZE did not induce zoosporic germination at 1/50 dilution, which was at the same concentration as ZE from 1 × 10^3^ spores/ml zoospore suspension. The liquid exudates before chilling and zoospore release did not have the same effects on zoospores of *P. erythroseptica*, which indicated that the signaling molecules were secreted, by the zoospores instead of by other structures of *P. erythroseptica* (Table 1). All those results illustrated that *P. erythroseptica* zoosporic germination was triggered by extracellular signal molecules released by zoospores themselves. CaCl_2_ started to induce zoosporic germination of *P. erythroseptica* at a concentration of 10 mM (Table 1). This concentration was higher than the 10^5^ fold of Ca^2+^ in ZE tested by Warburton and Deacon (1998). Neither V8 nor lemma bean broth induced zoosporic germination at the test concentration (Table 1).

**Table 1:** Effect of zoospore exudate (ZE) and other chemicals on zoosporic germination of *Phytophthora erythroseptica*.

Tested solution	Germination (%)	Standard deviation
Sterile distilled water	0.0 a	0.0
0.01 mM CaCl_2_	0.0 a	0.0
0.1 mM CaCl_2_	0.0 a	0.0
1 mM CaCl_2_	0.0 a	0.0
10 mM CaCl_2_	5.0 b	4.8
10^-4^ V8 broth	0.0 a	0.0
10^-4^ lemma bean broth	0.0 a	0.0
Zoospore exudate	38.1 d	7.7
Liquid exudates before chilling	0.0 a	0.0
Liquid exudates immediately after chilling	0.0 a	0.0
5 mM leucine	14.9 bc	5.2

*Zoospore suspension of *Phytophthora erythroseptica* was incubated in zoospore exudate, calcium chloride, lemma bean broth, or V8 broth. Different letters indicate significant difference (*P* < 0.05) among treatments detected by Fisher’s protected least significant difference.*

### Effect of ZE on Zoosporic Infection of *P. erythroseptica* on Potato Tuber

Low-density zoospore suspensions (5 zoospores/inoculation per site) did not cause symptom on tuber slice or whole tuber (Figure 3A1,A2). Zoospores at higher density (100 zoospores/inoculation per site) caused symptom on both tuber slice, as brown necrosis lesions, and on whole tuber as pink color on the cut surface (Figure 3C1,C2). However, when zoospore suspension was treated with ZE, even 5 zoospores could cause brown necrosis lesions on tuber slice and whole tuber (Figure 3B1,B2). The disease incidences had no difference between zoospores treated with ZE and non-treated zoospores at high density (*P* > 0.1) (data were not show).

### Characterization of ZE

Zoospore exudate was stable when treated with high temperature. Autoclaved ZE still induced zoosporic germination (Table 2). The germination ratio of zoospores in treated ZEs had no significant difference compared with untreated ZE, except ZE treated with 40°C, which had even higher induced activity (Table 2). Similar results were achieved in the tuber infection assay. The ZE activity was significantly reduced after treatment with proteinase K. However, catalase did not have any effect on ZE (Table 2).

**Table 2:** Effect of temperature and enzyme on the activity of *Phytophthora erythroseptica* zoospore exudate (ZE) on zoosporic germination at 500 spores/ml.

Treatment	Treating time (min)	Zoosporic germination (%)	Infection of potato tuber
Non-treated ZE	–	83.6 ± 17.2 b	Positive
40°C	20	98.0 ± 2.0 a	Positive
60°C	20	82.0 ± 6.9 b	Positive
80°C	20	88.8 ± 6.8 ab	Positive
100°C	20	94.7 ± 1.2 ab	Negative
121°C	30	93.6 ± 5.2 ab	Not tested
Proteinase K	120	41.0 ± 8.9 c	Not tested
Catalase	120	88.1 ± 8.6 ab	Not tested

*Different letters indicate significant difference (*P* < 0.05) among treatments detected by Fisher’s protected least significant difference.*

### Identification of Signal Compounds in ZE

The chromatogram of ZE analyzed by HPLC-DAD illustrated peaks at 3, 5, 10, 14, 15, and 16 min (Supplementary Figure S1A). Eluate fractions were collected separately into five groups at the following retention times: 2–5 min, 10–12 min, 12–14 min, 14–16 min, and 16–20 min. Compared with the control, fractions from time ranges of 12–14 min and 14–16 min induced zoosporic germination, with the 12–14 min fraction having the highest rate of germination (Supplementary Figure S1B). The eluate from 12 to 16 min was collected separately and further analyzed using LC-MS. Two amino acids were detected and identified as phenylalanine (Supplementary Figure S2A), leucine (Supplementary Figure S2B). Standard samples of phenylalanine and leucine were purchased and tested in the germination assay. The result showed that leucine induced zoosporic germination compared with control (Table 1). The length of zoospore germ tubes induced by leucine was not significantly different compared with control, which indicated that leucine induced zoosporic germination as a signaling molecule instead of as nutrient. However, phenylalanine did not have any effect on zoosporic germination.

## Discussion

It has been observed in several species of oomycetes that zoosporic behavior, such as homing responses, is driven by signal compounds (Deacon and Donaldson, 1993; Savory et al., 2014). This type of activity can be self-triggered or triggered by environmental factors (Deacon and Donaldson, 1993). In this study, we demonstrated that zoosporic germination and host infection of *P. erythroseptica* were density-dependent behaviors. In other words, zoospores at a concentration of higher than 1 × 10^3^ zoospores/ml could germinate, and germination did not occur when the concentration was bellow 1 × 10^3^ zoospores/ml. However, low density of *P. erythroseptica* zoospores did germinate if treated with ZE. Both zoosporic density and ZE directed toward the same mechanism: zoosporic germination was regulated by signal molecules. It seemed that there was a threshold of signaling molecule concentration at which zoosporic germination could be triggered. These results were in agreement with other studies in oomycetes (Kong and Hong, 2010).

Based on the LC-MS results, we identified that the amino acid leucine was related to zoosporic germination and therefore considered as a signal molecule. By testing serial dilutions of leucine, we found that 5 mM was the minimal concentration affecting zoosporic germination (Table 1). This concentration was similar to the levels of lysine reported by Byrt et al. (1982) (reference added to the manuscript) on the study of zoosporic behavior of *Phytophthora cinnamomi*. In addition, Jones et al. (1990) and Deacon and Donaldson (1993) discussed the chemicals and concentrations on the behavior of zoospores for several oomycetes. Therefore, the leucine concentration used in the experiment was physiological relevant.

We demonstrated that zoosporic germination was not significantly affected by nutrients or minerals. This suggested that these amino acid signal molecules play a role as autoinducers instead of just nutrients that promote zoosporic aggregation or germination. Although Ca^2+^ affects zoosporic behaviors, such as aggregation, germination, and encystment (von Broembsen and Deacon, 1997; Deacon and Saxena, 1998; Warburton and Deacon, 1998), it only works as a secondary messenger rather than directly triggering zoosporic behaviors (Joint et al., 2007; Kong and Hong, 2010; Hu et al., 2013). In this research, we confirmed that Ca^2+^ was not a major factor that triggered zoosporic behaviors for *P. erythroseptica*. Ca^2+^ induced zoosporic germination only at a concentration of 10 mM, which is more than 10^5^ times higher than that in ZE derived from the 10^6^ spores/ml suspension (Warburton and Deacon, 1998). The biological activity of ZE was not affected by high temperatures, which indicated the active compounds may not be proteins. However, proteinase K partially reduced ZE activity which indicated that the active molecules might be polypeptides. It was not determined how the two identified amino acids are related to polypeptides.

We found that zoosporic germination was originally regulated and mediated by extracellular signal molecules produced by zoospores of *P. erythroseptica*. More importantly, only the liquid exudates collected after zoospore release had this effect, which confirmed that those extracellular signal molecules were secreted by the zoospore itself instead of mycelia, sporangia, or other structures of *P. erythroseptica*.

There is also the possibility that the signal molecules are from other sources, such as soil microbes and plants. In other studies, zoosporic germination of several *Phytophthora* species was triggered by the presence of plant tissue, which indicated that plant exudates may contain either the same type or homologous compounds acting as signals (Dijksterhuis and Deacon, 2003; Kong and Hong, 2010; Suo et al., 2016). In a preliminary result, we showed that both soil microbes and plant root exudates provide similar function as the signal molecules (Jiang and Hao, 2015). Whether these chemicals are the same remains unknown currently, but this is our future objective.

Zoosporic auto-aggregation was observed in *P. palmivora, P. capsici, P. nicotianae, P. sojae*, and *P. infestans*, and was a required step before germination and was regulated and enhanced by threshold concentrations of extracellular signal molecules (Ko and Chan, 1974; Reid et al., 1995; Kong and Hong, 2010; Kong et al., 2010b; Savory et al., 2014). Kong et al. illustrated that extracellular molecules of zoospores mediated auto-aggregation, and high dosage of molecules can even trigger auto-aggregation of low density zoospores (Kong and Hong, 2010). However, no specific aggregation was observed in this study. These results suggest that *P. erythroseptica* may not need aggregation for germination, as needed in other *Phytophthora* species.

This research improved our understanding of the initiation and development of potato pink rot. More broadly, we may be able to provide insight into microbial ecological systems with multi-faceted communications. Based on this mechanism, novel strategies of pink rot management may be developed by applying chemicals that either suppress or promote the cell-to-cell communication of zoospores.

## Author Contributions

HJ was responsibility of experimental design, data collection and analysis, and manuscript writing. HWH contributed to experimental design, data collection, and manuscript modification for the chemical identification. TG contributed to experimental design and data collection for the chemical identification. BC contributed to equipments, experimental design, and manuscript modification. BP provided the equipments for chemical analysis. JH supervised the research project.

## Conflict of Interest Statement

The authors declare that the research was conducted in the absence of any commercial or financial relationships that could be construed as a potential conflict of interest.

## Supplementary Material

FIGURE S1 Effects of chemicals separated from zoospore exudate (ZE) of *Phytophthora erythroseptica* using high performance liquid chromatograph analysis (HPLC). Chemical peaks shown on the chromatograph at different retention time **(A)**. Germination of zoospores were examined after treated with water or collected chemicals from HPLC according to retention time **(B)**. Different letters indicate significant difference (*P* < 0.05) among treatments detected by Fisher’s protected least significant difference.

FIGURE S2 Mass spectrum of peaks eluted from residua of 12–16 min in HPLC. Peak at *m/z* 165.7 **(A)** produced the MS^2^ base peak at *m/z* 119.9, which was the same as phenylalanine (the structure was shown). Peak at *m/z* 132.1 **(B)** produced the MS^2^ base peak at *m/z* 86.3, which was the same as leucine (the structures were shown).

## References

[B1] Byrt P. N. Irving H. R. Grant B. R. (1982). The effect of organic compounds on the encystment, viability and germination of zoospores of *Phytophthora cinnamomi*. *J. Gen. Microbiol.* 128 2343–2351. 10.1099/00221287-128-10-2343

[B2] Chapara V. Taylor R. J. Pasche J. S. Gudmestad N. C. (2011). Competitive parasitic fitness of mefenoxam-sensitive and -resistant isolates of *Phytophthora erythroseptica* under fungicide selection pressure. *Plant Dis.* 95 691–696. 10.1094/PDIS-10-10-0730 30731895

[B3] Chapara V. Taylor R. J. Pasche J. S. Secor G. A. Gudmestad N. C. (2007). Fungicide efficacy for control of mixed populations of *Phytophthora erythroseptica*. *Phytopathology* 97:S160.

[B4] Clarke D. D. (1966). Factors affecting the development of single zoospore colonies of *Phytophthora infestans*. *Trans. Br. Mycol. Soc.* 49 177–184. 10.1016/S0007-1536(66)80051-7

[B5] Deacon J. W. Donaldson S. P. (1993). Molecular recognition in the homing responses of zoosporic fungi, with special reference to *Pythium* and *Phytophthora*. *Mycol. Res.* 97 1153–1171. 10.1016/S0953-7562(09)81278-1

[B6] Deacon J. W. Saxena G. (1998). Germination triggers of zoospore cysts of *Aphanomyces euteiches* and *Phytophthora parasitica*. *Mycol. Res.* 102 33–41. 10.1017/S0953756297004358

[B7] Dijksterhuis J. Deacon J. W. (2003). Defective zoospore encystment and suppressed cyst germination of *Phytophthora palmivora* caused by transient leaching treatments. *Antonie Van Leeuwenhoek Int. J. Gen. Mol. Microbiol.* 83 235–243. 12776919 10.1023/a:1023360326015

[B8] Erwin D. C. Ribeiro O. K. (1996). *Phytophthora Diseases Worldwide.* St. Paul, MN: APS Press.

[B9] Fitzpatrick-Peabody E. (2011). Methodology and assessment of the susceptibility of potato genotypes to *Phytophthora erythroseptica* causal prganism of pink rot. *Am. J. Potato Res.* 88 105–113. 10.1007/s12230-010-9179-7

[B10] Fuqua C. Parsek M. R. Greenberg E. P. (2001). Regulation of gene expression by cell-to-cell communication: acyl-homoserine lactone quorum sensing. *Annu. Rev. Genet.* 35 439–468. 10.1146/annurev.genet.35.102401.090913 11700290

[B11] Hirsch A. M. Bauer W. D. Bird D. M. Cullimore J. Tyler B. Yoder J. I. (2003). Molecular signals and receptors: controlling rhizosphere interactions between plants and other organisms. *Ecology* 84 858–868. 10.1890/0012-9658(2003)084[0858:MSARCR]2.0.CO;2

[B12] Hornby J. M. Jensen E. C. Lisec A. D. Tasto J. J. Jahnke B. Shoemaker R. (2001). Quorum sensing in the dimorphic fungus *Candida albicans* is mediated by farnesol. *Appl. Environ. Microbiol.* 67 2982–2992. 10.1128/AEM.67.7.2982-2992.2001 11425711 PMC92970

[B13] Hu L. Wang D. Liu L. Chen J. Xue Y. Shi Z. (2013). Ca^2+^ efflux is involved in cinnamaldehyde-induced growth inhibition of *Phytophthora capsici*. *PLoS One* 8:e76264. 10.1371/journal.pone.0076264 24098458 PMC3788004

[B14] Jiang H. Hao J. (2015). Behaviorof *Phytophthora erythroseptica* in plant infection mediated by microbe-secreted compounds. *Phytopathology* 105:S1.7.

[B15] Joint I. Tait K. Wheeler G. (2007). Cross-kingdom signalling: exploitation of bacterial quorum sensing molecules by the green seaweed Ulva. *Philos. Trans. R. Soc. B Biol. Sci.* 362 1223–1233. 10.1098/rstb.2007.2047 17360272 PMC2435585

[B16] Jones S. W. Donaldson S. P. Deacon J. W. (1990). Behaviour of zoospores and zoospore cysts in relation to root infection by *Pythium aphanidermatum*. *New Phytol.* 117 289–301. 10.1111/j.1469-8137.1991.tb04910.x

[B17] Ko W. H. Chan M. J. (1974). Aggregation of *Phytophthora capsici* zoospores and their interaction with zoospores of *P. palmivora*. *Microbiology* 80 549–551. 10.1099/00221287-80-2-549

[B18] Kong P. Hong C. (2010). Zoospore density-dependent behaviors of *Phytophthora nicotianae* are autoregulated by extracellular products. *Phytopathology* 100 632–637. 10.1094/PHYTO-100-7-0632 20528180

[B19] Kong P. Hong C. X. (2016). Soil bacteria as sources of virulence signal providers promoting plant infection by *Phytophthora* pathogens. *Sci. Rep.* 6:33239. 10.1038/srep33239 27616267 PMC5018965

[B20] Kong P. Lee B. W. K. Zhou Z. S. Hong C. (2010a). Zoosporic plant pathogens produce bacterial autoinducer-2 that affects *Vibrio harveyi* quorum sensing. *FEMS Microbiol. Lett.* 303 55–60. 10.1111/j.1574-6968.2009.01861.x 20002192 PMC2891589

[B21] Kong P. Tyler B. M. Richardson P. A. Lee B. W. K. Zhou Z. S. Hong C. (2010b). Zoospore interspecific signaling promotes plant infection by *Phytophthora*. *BMC Microbiol.* 10:313. 10.1186/1471-2180-10-313 21138563 PMC3016323

[B22] Lambert D. H. Salas B. (1994). Metalaxyl insensitivity of *Phytophthora erythroseptica* isolates causing pink rot of potato in Maine. *Plant Dis.* 78:10 10.1094/PD-78-1010B

[B23] Li Z. Nair S. K. (2012). Quorum sensing: how bacteria can coordinate activity and synchronize their response to external signals? *Protein Sci.* 21 1403–1417. 10.1002/pro.2132 22825856 PMC3526984

[B24] Lonsdale D. Cunliffe C. Epton H. A. S. (1980). Possible routes of entry of *Phytophthora erythroseptica* Pethyb. and its growth within potato plants. *Phytopathologische Zeitschrift J. Phytopathol.* 97 109–117. 10.1111/j.1439-0434.1980.tb03677.x

[B25] Morris B. M. Reid B. Gow N. A. R. (1995). Tactic response of zoospores of the fungus *Phytophthora palmivora* to solutions of different pH in relation to plant infection. *Microbiology* 141 1231–1237. 10.1099/13500872-141-5-1231 33820117

[B26] Peters R. D. Sturz A. V. Matheson B. G. Arsenault W. J. Malone A. (2001). Metalaxyl sensitivity of isolates of *Phytophthora erythroseptica* in Prince Edward Island. *Plant Pathol.* 50 302–309. 10.1046/j.1365-3059.2001.00566.x

[B27] Pratt R. G. (1981). Morphology, pathogenicity, and host range of *Phytophthora megasperma, Phytophthora erythroseptica*, and *Phytophthora parasitica* from arrowleaf clover. *Phytopathology* 71 276–282. 10.1094/Phyto-71-276

[B28] Raina S. De Vizio D. Palonen E. K. Odell M. Brandt A. M. Soini J. T. (2012). Is quorum sensing involved in lovastatin production in the filamentous fungus *Aspergillus terreus*? *Process Biochem.* 47 843–852. 10.1016/j.procbio.2012.02.021

[B29] Raina S. Odell M. Keshavarz T. (2010). Quorum sensing as a method for improving sclerotiorin production in *Penicillium sclerotiorum*. *J. Biotechnol.* 148 91–98. 10.1016/j.jbiotec.2010.04.009 20438771

[B30] Reid B. Morris B. M. Gow N. A. R. (1995). Calcium-dependent, genus-specific, autoaggregation of zoospores of Phytopathogenic fungi. *Exp. Mycol.* 19 202–213. 10.1006/emyc.1995.1025

[B31] Savory A. I. M. Grenville-Briggs L. J. Wawra S. Van West P. Davidson F. A. (2014). Auto-aggregation in zoospores of *Phytophthora infestans*: the cooperative roles of bioconvection and chemotaxis. *J. R. Soc. Interface* 11 1–8. 10.1098/rsif.2014.0017 24598206 PMC3973368

[B32] Suo B. Chen Q. M. Wu W. X. Wu D. Tian M. Jie Y. (2016). Chemotactic responses of *Phytophthora sojae* zoospores to amino acids and sugars in root exudates. *J. Gen. Plant Pathol.* 82 142–148. 10.1007/s10327-016-0651-1

[B33] Taylor R. J. Pasche J. S. Gudmestad N. C. (2011). Effect of application method and rate on residual efficacy of mefenoxam and phosphorous acid fungicides in the control of pink rot of potato. *Plant Dis.* 95 997–1006. 10.1094/PDIS-09-10-0694 30732101

[B34] Taylor R. J. Salas B. Secor G. A. Rivera V. Gudmestad N. C. (2002). Sensitivity of north American isolates of *Phytophthora erythroseptica* and *Pythium ultimum* to mefenoxam (metalaxyl). *Plant Dis.* 86 797–802. 10.1094/PDIS.2002.86.7.797 30818580

[B35] Vargas L. A. Nielsen L. W. (1972). *Phytophthora erythroseptica* in Peru: ITS identification and pathogenesis. *Am. Potato J.* 49 309–320. 10.1007/BF02861669

[B36] von Broembsen S. L. Deacon J. W. (1997). Calcium interference with zoospore biology and infectivity of *Phytophthora parasitica* in nutrient irrigation solutions. *Phytopathology* 87 522–528. 10.1094/PHYTO.1997.87.5.522 18945107

[B37] Warburton A. J. Deacon J. W. (1998). Transmembrane Ca^2+^ fluxes associated with zoospore encystment and cyst germination by the phytopathogen *Phytophthora parasitica*. *Fung. Genet. Biol.* 25 54–62. 10.1006/fgbi.1998.1086 9806806

