# Supplementary figures and images for: Leucine Regulates Zoosporic Germination and Infection by Phytophthora erythroseptica
Source: Front Microbiol. 2019 Feb 5;10:131. doi: 10.3389/fmicb.2019.00131 (PMC6370700; doi:10.3389/fmicb.2019.00131)

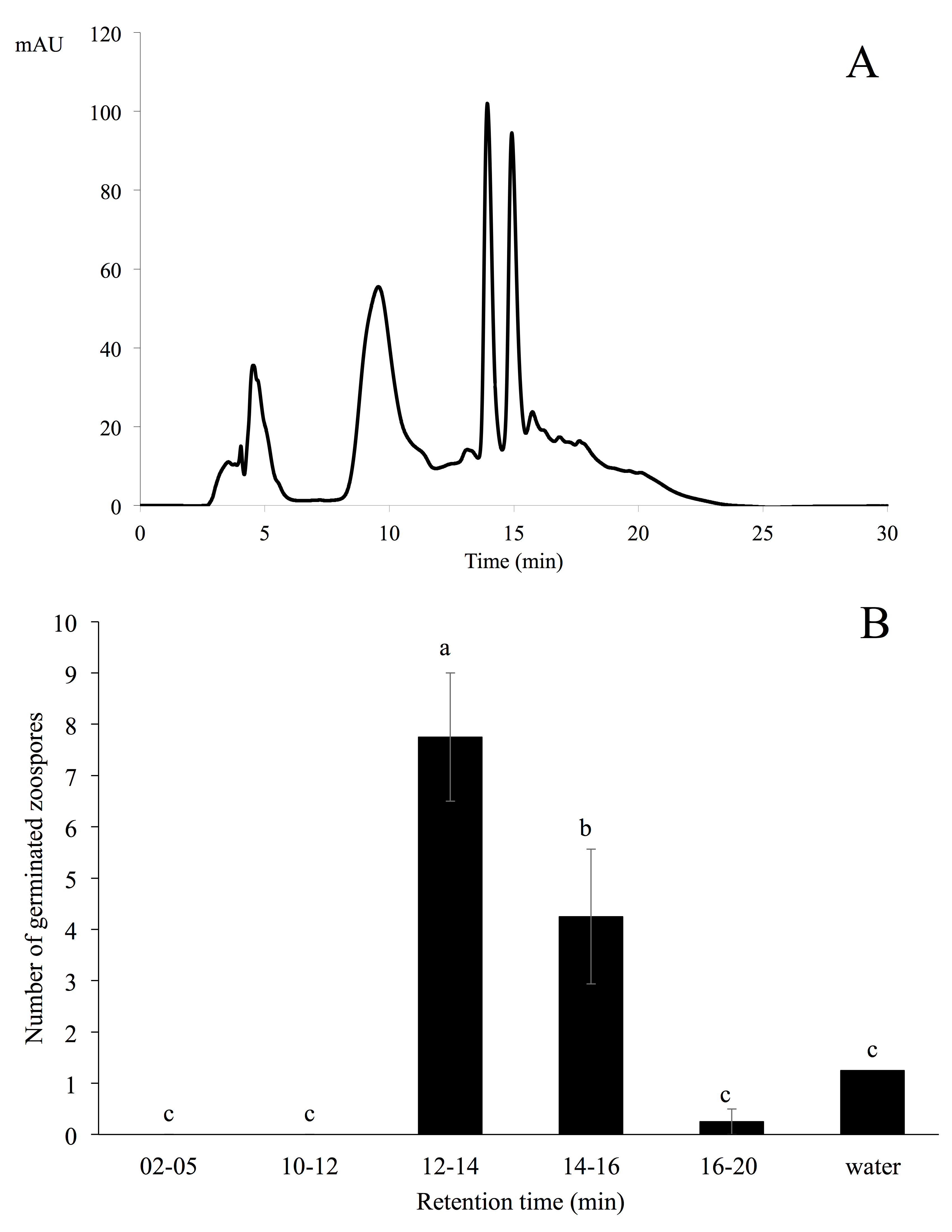

Supplement: FIGURE S1 — Effects of chemicals separated from zoospore exudate (ZE) of Phytophthora erythroseptica using high performance liquid chromatograph analysis (HPLC). Chemical peaks shown on the chromatograph at different retention time (A). Germination of zoospores were examined after treated with water or collected chemicals from HPLC according to retention time (B). Different letters indicate significant difference (P < 0.05) among treatments detected by Fisher’s protected least significant difference. [file Image_1.JPEG]

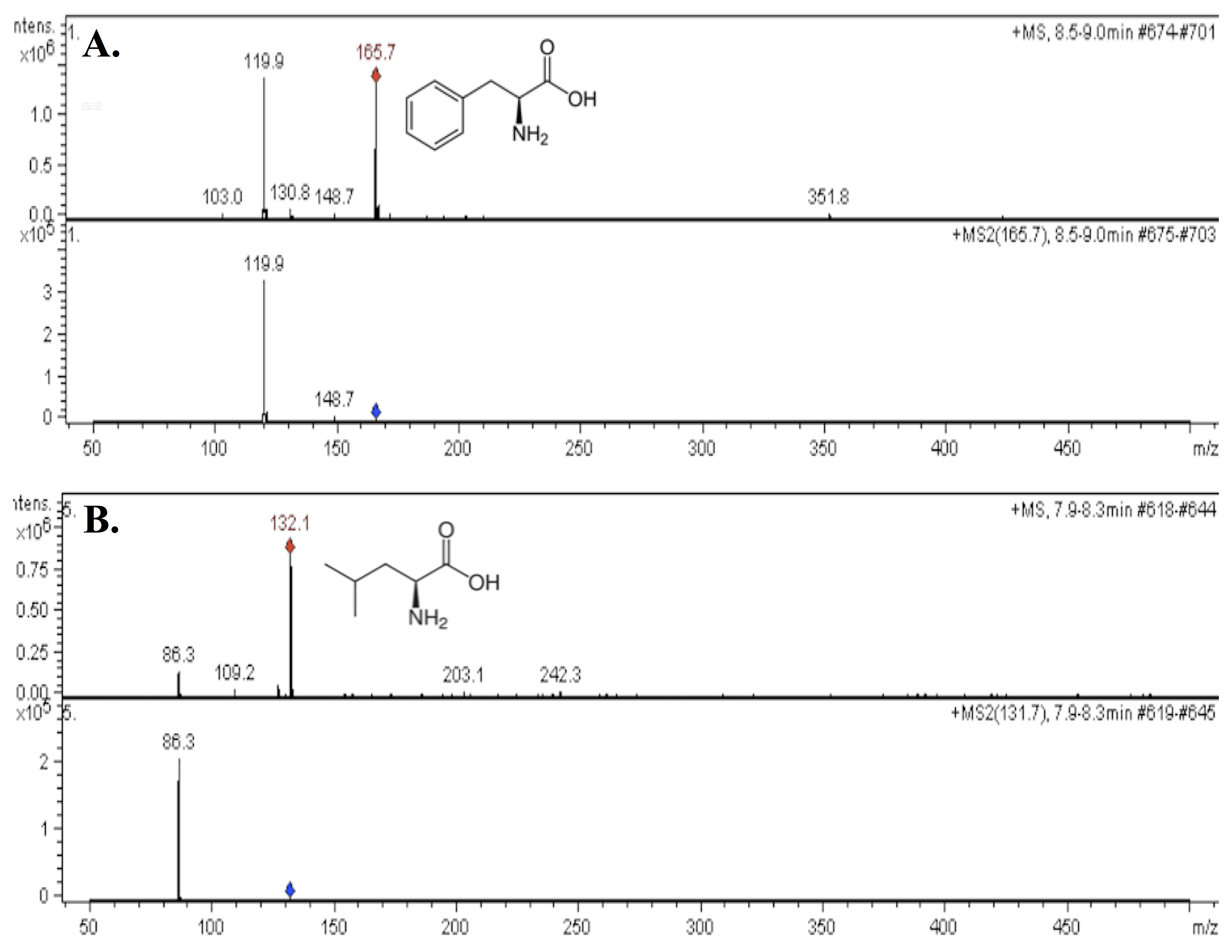

Supplement: FIGURE S2 — Mass spectrum of peaks eluted from residua of 12–16 min in HPLC. Peak at m/z 165.7 (A) produced the MS2 base peak at m/z 119.9, which was the same as phenylalanine (the structure was shown). Peak at m/z 132.1 (B) produced the MS2 base peak at m/z 86.3, which was the same as leucine (the structures were shown). [file Image_2.JPEG]
